# Looking Through “Rose-Tinted” Glasses: The Influence of Tint on Visual Affective Processing

**DOI:** 10.3389/fnhum.2019.00187

**Published:** 2019-06-06

**Authors:** Tim Schilling, Alexandra Sipatchin, Lewis Chuang, Siegfried Wahl

**Affiliations:** ^1^Institute for Ophthalmic Research, Eberhard Karls University Tuebingen, Tuebingen, Germany; ^2^Department of Perception, Cognition and Action, Max Planck Institute for Biological Cybernetics, Tuebingen, Germany; ^3^Department of Human-Centered Ubiquitous Media, Institute for Informatics, Ludwig-Maximilians-University Munich, Munich, Germany; ^4^Carl Zeiss Vision International GmbH, Aalen, Germany

**Keywords:** electroencephalography (EEG), physiological response, color-tinted lenses, red, emotion

## Abstract

The use of color-tinted lenses can introduce profound effects into how we process visual information at the early to late stages. Besides mediating harsh lighting conditions, some evidence suggests that color-tinted lenses can influence how humans respond to emotional events. In this study, we systematically evaluated how color-tinted lenses modified our participants’ psychophysiological responses to emotion-inducing images. The participants passively viewed pleasant, neutral or unpleasant images from the International-Affective-Picture-System (IAPS), while wearing none, blue, red, yellow or green tinted-lenses that were controlled for luminance. Established neuroergonomic indices of arousal were measured on the autonomic level, namely Skin-Conductance-Response (SCR) and Heart-Rate-Variability (HRV), and on the cortical level, with electroencephalography (EEG) event-related potentials (ERPs). Phasic SCR responses were significantly enhanced for unpleasant images and both pleasant and unpleasant images induced significantly larger ERP amplitudes of the Late-Positive-Potential (LPP), with pleasant images having the greatest impact. Interestingly, a significant main effect was found for tint. Similar to viewing pleasant images, red-tinted lenses induced the largest LPPs. Taken together, these findings suggest that the autonomic response to affective images is modulated at the cortical level of processing, congruent with the use of red-tinted lenses.

## Introduction

It is claimed that soldiers of the US Civil War were prescribed glasses with colored lenses with the intention to treat disorders such as depression (Henry, 2016). Supposedly this practice gave rise to the phrase “To see the world through rose colored glasses,” which refers to individuals who hold a positive outlook on life. Regardless of the veracity of this apocryphal tale, color is an important ubiquitous feature of the visual world that the brain is highly selective for. It allows us to readily recognize objects (Gegenfurtner and Rieger, 2000), facilitates visual memory (Wichmann et al., 2002) and can be used to segment objects from the background (Gegenfurtner, 2003). Color can also exert a strong influence on physiology.

Blue light has an essential function in chronobiology as a pacemaker of the circadian rhythm (Berson et al., 2002). It mediates the circadian rhythm through intrinsically photosensitive retinal ganglion cells containing the photopigment melanopsin, which is most sensitive to approximately 480 nm (Berson et al., 2002). Green light has been found to exacerbate migraine headache less than white, blue, amber or red lights (Noseda et al., 2016). Red coloration is associated with male dominance and testosterone in animals (Andersson et al., 2002; Pryke et al., 2002; Setchell and Jean Wickings, 2005) and it may reflect dominance in humans as well (Hill and Barton, 2005). For example, human anger correlates with reddening of the skin due to increased blood flow (Darwin and Prodger, 1998; Drummond and Quah, 2001). It has been proposed, that color associations and preferences developed on the basis of emotions evoked by colored objects (Ou et al., 2004a,b). The ability to detect red colored objects such as ripe fruits and berries against green leaves was evolutionarily important. It has been claimed that the color vision system adapted its color preferences, especially in female (Hurlbert and Ling, 2007). The hypothesis that colors have ecological value is summarized in the Ecological Valence Theory (Palmer and Schloss, 2010).

Colors can also strongly influence emotional processing. According to the circumplex model, emotions can be classified into two categories: affective valence (pleasure-displeasure axis) and arousal (arousal-sleepiness axis; Russell, 1980); these categories showed a clear relation to physiological responses (Lang et al., 1993; Mauss and Robinson, 2009). The color red is connected with excitement (Wexner, 1954) and can increase arousal i.e., greater electroencephalography (EEG) alpha wave recovery (Ali, 1972) and Galvanic-Skin-Response (GSR; Wilson, 1966; Jacobs and Hustmyer, 1974; Lee and Westland, 2015). Besides physiological arousal, red is also linked to emotions on a color association level. It has positive appetitive implications like attraction (Elliot and Niesta, 2008). On the opposite, as a negative valence color, red is linked to danger, failure (Gerend and Sias, 2009), anger and aggression (Elliot and Maier, 2007; Fetterman et al., 2012). For example, a red background makes it easier to categorize angry faces (Young et al., 2013). This influence on psychological functioning, emotional and cognitive decision-making is known as the red effect (Gilston and Privitera, 2016). Green, as the opponent color to red, is often connected to a positive meaning (Elliot et al., 2007; Gnambs et al., 2010) e.g., green promotes creativity (Lichtenfeld et al., 2012) and is associated with safety (Pravossoudovitch et al., 2014). In addition, green hue is said to have a calming, stress-reducing effect (Jalil et al., 2012). Going out to the natural environment with plants containing chlorophyll is associated with green as well (Akers et al., 2012). Yellow hue is often used for warning signs in combination with black. In nature, this warning sign of yellow-black contrast appears on bees for instance (Rowe and Halpin, 2013).

Color can be seen naturally or it can be modified on a spectral illumination level. The modification of visual light occurs already in the atmosphere for sun light and generally when absorbed or reflected from any object. The light, which is reflected or emitted by the object, appears as the object color. Almost every object is emitting or reflecting a certain wavelength pattern, called spectrum. Filters can modify these spectra through wavelength-specific reflection, absorption and transmission. Neutral density or gray filters reduce the spectra equally over all wavelengths, whereas tinted lenses selectively absorb the wavelengths except for those related to the tint. For instance, a red-tinted lens transmits light higher than ca. 600 nm and absorbs light below. Tinted lenses are also called filters or colored glasses. A common filter is the yellow cut-off filter, which is a high pass filter, strongly absorbing light of wavelengths lower than the cut-off wavelength (ca. 480–500 nm) and transmitting light of higher wavelengths than this cut-off wavelength. From a cognitive point of view, yellow-tinted lenses have been shown to modulate the level of attention: reading speed (Hollingsworth et al., 2015) and response time (Lacherez et al., 2013) were improved by yellow tints. From a physiological point of view, yellow-tinted lenses increase the pupils size (Kelly, 1990; Chung and Pease, 1999) and prevent short wavelength light from reaching the eye, protecting (Downie, 2017; Lawrenson et al., 2017) the eye from light damage similarly like the UV-blocking function (International Commission on Non-Ionizing Radiation Protection, 2004). It is important to keep in mind that color and colored light modulated by tinted lenses are not necessarily the same. For example, a color appears often strong when there is a color contrast, whereas colored filters alter the spectrum that is transmitted to the eye and color contrast is then usually diminished. The strongest difference between color and colored light appears when colored light is filtered completely to a monochromatic narrow wavelength bandwidth by tinted lenses. Under this condition, no color contrast will be presented on the retina. Limitations of previous studies have been the unequal luminance levels for different color conditions. In this study, we carefully calibrated the luminance resulting in equal luminance for each tinted lens condition.

It has already been reported that there are abundant studies on the colors red and blue and that other colors are underrepresented (Jalil et al., 2012). Despite such extensive research into color, so far no investigations are known to the authors on the effect of tinted lenses on visual affective processing. Transferring the results from psychological color science to optically tinted lenses, one could expect that tinted lenses influence how humans respond to emotional events. The purpose of this study was to investigate such a potential influence of color-tinted lenses on visual affective processing at both the autonomic level, meaning heart-rate-variability (HRV) and skin-conductance-response (SCR) measures, as well as the cortical level, meaning EEG measures. In particular, it is predicted that images with emotional content will give rise to larger responses at both levels. If color-tint has an influence, it ought to moderate these responses, especially at the cortical level. As a sustained positive component of the event-related potential (ERP) waveform in the EEG, the LPP indicates a selective processing of emotional stimuli and activation of motivational systems in the brain (Cuthbert et al., 2000); it is a well-established finding, that emotional arousing pictures induce a more positive LPP than neutral pictures (Cuthbert et al., 2000; Schupp et al., 2000; Hajcak et al., 2009, 2010; Brown et al., 2012). The focus was on the LPP component for two reasons: color is related to emotional processing (Jalil et al., 2012), and, the LPP component reflects emotional arousal level (Cuthbert et al., 2000; Schupp et al., 2000; Dolcos and Cabeza, 2002; Spreckelmeyer et al., 2006). Among the plenty of studies on emotions and LPPs, previous work has shown that a non-meditating control group had higher LPP compared to meditators while viewing unpleasant International-Affective-Picture-System (IAPS) stimuli (Sobolewski et al., 2011). Regarding emotional regulation, the LPP is reduced when emotions are strategically suppressed (Moser et al., 2006; Foti and Hajcak, 2008). Additionally, LPP reflects automatic attention to emotional visual stimuli (Hajcak et al., 2009). A review about the relation of affective picture processing and ERP components reveals that attention is linked to early components (<300 ms) and memory encoding is associated with later components (>300 ms; Olofsson et al., 2008); besides LPP, also other ERP components as the P300 are related to emotional stimuli (Hajcak et al., 2010). The LPP was selected to be investigated, because of its often-described connection to arousal, motivation and attention, when affective pictures are presented, in the literature.

Autonomic responses were evaluated in terms of HRV and SCR responses. It has been shown that the heart rate co-varies with affective valence, whereas SCR was increased with arousal (Cuthbert et al., 2000). These previous findings are validated here, namely that phasic SCR was significantly larger for unpleasant images compared to neutral images.

## Materials and Methods

Three different physiological responses were assessed: EEG, HRV and SCR.

### Participants

Thirty-one participants were enrolled in this study, which contained 12 male and 19 female participants. The average age was 25.9 ± 3.5 years and the objective refractive errors were corrected to normal vision using trial lenses (mean spherical refractive error: OD −2.80 ± 2.37 D, OS −2.55 ± 2.28 D) measured with an open field autorefractor (Grand Seiko WAM-5500, Grand Seiko Co., Ltd., Fukuyama Hiroshima, Japan). The study followed the tenets of the Declaration of Helsinki and was approved by the Institutional Review Board of the Medical Faculty of the University of Tuebingen. Informed consent was obtained from all participants after the content and possible consequences of the study had been explained.

### Stimuli and Experimental Procedure

**Figure 1:**
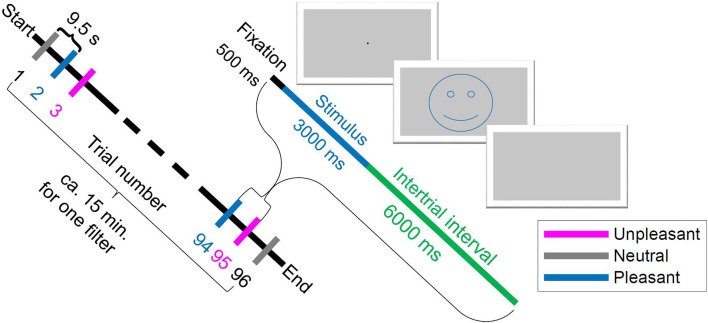
Scheme of experiment procedure: one trial began with 500 ms fixation, then 3 s stimulus image presentation and an inter-trial interval of 6 s. The smiley just illustrates the valence and was not shown in the experiment. The order of trial numbers from Start to End is an example of a randomized order.

For this study, stimuli from the IAPS were used with normalized ratings for valence and arousal level to experience emotional events (Lang et al., 2008). According to Russell’s valence-arousal model (Russell, 1980), stimuli are usually separated into non-emotional (neutral) stimuli with low arousal and emotional (pleasant, unpleasant) stimuli with high arousal (e.g., Keil et al., 2002). Stimuli consisted of 96 pictures from the IAPS (Lang et al., 2008), with 32 pleasant^1^ (adventure, erotic, sport, babies; mean pleasure = 7.0; mean arousal: 5.5), 32 neutral^2^ (people; mean pleasure = 4.9; mean arousal = 3.6), and 32 unpleasant^3^ (mutilated bodies, guns, sad; mean pleasure = 2.4; mean arousal = 5.9) pictures. The experimental design was adapted from Bradley et al. (2008), as detailed next, to avoid disturbances from unbalanced self-reported arousal and visual complexity of the images. This study involved five different tint-filtering conditions. Each filter condition was tested in 16 blocks of trials, with each block containing two neutral, two unpleasant and two pleasant images in random order. One block thus contained six trials, resulting in 96 trials per tint. Each trial consisted of 500 ms fixation period, 3 s stimulus presentation and 6 s inter-trial-interval, as illustrated in Figure 1. The tint order was also randomized to avoid LPP potentiation for the repeated images for each tint presentation (Codispoti et al., 2007). Pictures were presented using the Psychophysics Toolbox Version 3.0.11 (Kleiner et al., 2007) under MATLAB (Matlab R2013B, 64 bit, MathWorks Inc., Natick, MA, USA) running on a PC with Windows 7 Enterprise, 64 bit operating system. Participants passively viewed the images presented on a ViewPixx 3D LCD-Display (ViewPixx 3D, VPixx Technologies Inc., Saint-Bruno, QC, Canada) with 16 bit grayscale resolution at a distance of 1 m. The images subtended a visual angle of 16° horizontally and 12° vertically. The mean luminosity across all grayscale images was computed and the contrast of each single image was adjusted so that the sum of luminance values across all pixels resulted in the mean luminosity value. Interestingly, if the color information of emotional pictures is removed, this had no effect on affective modulation in LPP (Codispoti et al., 2012). Therefore, a difference is not expected when using grayscale instead of colored images. Thus, this study controlled for color information within the images themselves—all pictures were presented in grayscale. If color information was available, effects from local color contrasts could not be excluded, which might vary from image to image. Participants wore artificially tinted lenses that colored the entire grayscale picture homogenously and did not selectively filter image contents locally. Although the majority of human visual experience is not in grayscale, but rather naturally or artificially lit, gray-scaling has the advantage of investigating the effect of color-tinted lenses only rather than effects of local color differences.

### Color-Tinted Lenses

**Figure 2:**
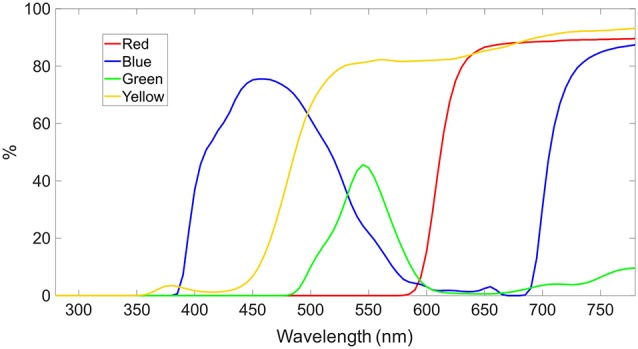
Spectral transmission of color-tinted lenses in percentage. The yellow- and red-tinted lens are high-pass filter with cut-off at ca. 480 nm and 610 nm, respectively. The blue- and green-tinted lens transmits the spectral curve of an inverted U-shape with peak at ca. 460 nm and 550 nm, respectively.

The tinted lenses included a blue-tinted lens (Carl Zeiss Vision Italia SpA, Varese, Italia), a red-tinted lens (Carl Zeiss Vision Italia SpA, Varese, Italia), a green-tinted lens (Carl Zeiss Vision Italia SpA, Varese, Italia) and a yellow-tinted lens (Carl Zeiss Vision International GmbH, Aalen, Germany). Transmission spectra are plotted in Figure 2. In the none-tinted lens condition, the participants wore only the trial lenses to be corrected to normal vision.

The luminance of the ViewPixx monitor was adjusted for each tinted lens, by a spectrometer (SpecWin Pro spectrometer, Instruments Systems GmbH, Munich, Germany) to 2.40 ± 0.07 cd/m^2^. For comparison with other studies, CIE color coordinates were measured as: no filter/none (*x* = 0.25, *y* = 0.25, *z* = 0.50), blue (*x* = 0.13, *y* = 0.19, *z* = 0.68), yellow (*x* = 0.34, *y* = 0.42, *z* = 0.24), green (*x* = 0.17, *y* = 0.74, *z* = 0.08) and red (*x* = 0.60, *y* = 0.29, *z* = 0.12) tinted lens.

### Preference

Participants were asked to evaluate their preference for each tinted lens just before the experimental tint condition started (main experiment). For each lens, they selected one out of three options: like (preferred), dislike (not preferred) or neutral. This approach was not suitable for statistical analysis; therefore, absolute values in percentage were described.

### Heart Rate Variability

HRV was measured using a photoelectric pulse sensor (g.PULSEsensor, g.tec medical engineering GmbH, Schiedlberg, Austria). The sensor was positioned at the tip of the index finger and monitored analog pulse wave signal changes in the reflected blood vessels’ light (0.01–60 Hz). HRV was analyzed by calculating the frequency-domain measure low-filter/high-filter (LF/HF) ratio with the HRVAS analysis tool (Ramshur, 2010).

### Galvanic Skin Response

Skin conductance, also called electro-dermal activity was measured using a GSR sensor (g.GSRsensor, g.tec medical engineering GmbH, Schiedlberg, Austria). Two small electrodes were fixed on the third and the fourth finger through a wrapping band. The data was decomposed into its phasic and tonic component using the Ledalab toolbox (Benedek and Kaernbach, 2010). First, it was downsampled to 20 Hz and then processed by the Continuous Decomposition Analysis. For SCR, the integral of the phasic activity over the response window (ISCR) was taken in a time window of 1–9 s after stimulus onset (Benedek and Kaernbach, 2010). While this phasic driver is linked to the event related SCR responses, the tonic activity characterizes the basic SCR level. To correct for skewed data distribution, SCR data was log10-transformed (Boucsein, 2012) resulting in normalization.

### Electroencephalography

EEG has become popular to address emotional processing in the human brain (Brown et al., 2012). In relation to EEG, the focus was on a particular component, the late positive potential (LPP), a slow change with a positive shift which can begin around 200–300 ms (Cuthbert et al., 2000; Schupp et al., 2000) or later around 500 ms (Dolcos and Cabeza, 2002; Spreckelmeyer et al., 2006). To record the EEG, 61 active electrodes (ActiCHamp, Brain Products GmbH, Gilching, Germany) were used based on the International 10/20 system held on the scalp with an elastic cap. The contact to the skin was ensured by gelling the electrodes to ensure low impedance (<20 kOhm). There was no gelling afterward. Four electrodes were used to record the Electrooculogram (EOG): the electrodes were positioned at the outer canthi of the left and right eye, and above and below the left eye. The central electrodes were placed vertically in a line with the nose from the forehead to the neck in the following order according to the 10/20 system: pre-frontal (Fp), anterior-frontal (AF), frontal (F), frontal-central (FC), central (C), central-parietal (CP), parietal (P), parietal-occipital (PO) and occipital (O). The reference electrode was fixed to the FCz and the ground electrode was placed at AFz. To localize the position in space individually, cap-tracking was performed using the CapTrak system Version 1.0 (Brain Products GmbH, Gilching, Germany) for 61 channels. The EOGs’ positions were set to default values. A differential amplifier system amplified the signal, which was then transferred to a data recording computer to record and store the EEG-data. Event triggers were sent from the display computer to the data recording computer *via* an analog/serial port to ensure synchrony of display and recorded data.

BrainVision Recorder Professional (V. 1.20.0701, Brain Products GmbH, Gilching, Germany) recorded the EEG-data with a sampling rate of 1,000 Hz, a sampling Interval of 1,000 μS, a low cut-off of 10 s (0.016 Hz), a high cut-off at 1,000 Hz, a resolution of 0.1 μV for EEG and 0.5 μV for EOG, Pulse and GSR. Although the signals of GSR and pulse sensor were differently amplified compared to EEG, all signals (Puls, GSR and EEG) were recorded in synchronization with the BrainVision Recorder. In offline processing, the data was downsampled to 250 Hz and high-pass filtered at 0.1 Hz with a basic finite impulse response (FIR) filter. Line noise was removed using the Matlab “CleanLine” function from Tim Mullen. After cleaning the data, offline re-referencing to the common average reference was performed. Artifact Subspace Reconstruction was used to correct EEG data, followed by an Adaptive Mixture Independent Component Analysis (AMICA) and a single equivalent current dipoles estimation for removing the biological artifacts. Data were then back-projected to the sensor space.

The ERP signal was processed by 200 ms pre-stimulus reference period and 3 s after picture onset. These 3 s are exactly the stimulus presentation duration. The reference was computed over all EEG electrodes, see common average reference. The late positive potential (LPP) between 500 ms and 1,500 ms was transmitted to statistical analysis. Processing and analysis of the ERP signal were performed with MATLAB and the open source MATLAB toolboxes EEGLAB Version 14.0.0b (Delorme and Makeig, 2004) and ERPLAB Version 6.1.3 (Lopez-Calderon and Luck, 2014).

Central electrodes were analyzed such as Fpz, AFz (mean of AF3 and AF4), Fz, FCz (mean of FC1 and FC2), Cz, CPz, Pz, PO and Oz. Channels Cz and FCz were chosen for detailed evaluation because both channels showed significant main effects for factor tint along the central line, see Table 1. In addition, the enhanced LPP for emotional stimuli relative to neutral shows its maximum in the centroparietal topography (Liu et al., 2012). To avoid movement related artifacts in EEG, pulse and GSR, the participants were instructed to refrain from moving during the recording session lasting for about 15 min.

**Table 1:** Analysis of variance (ANOVA) of Late-Positive-Potential (LPP) (500–1,500 ms) for central electrodes.

	Valence				Tint			Interaction		
	*df*	*F*	*p*	*ω*^2^	*F*	*p*	*ω*^2^	*F*	*p*	*ω*^2^
Fpz	4	8.12	<0.001	0.18	0.74	0.57	0.00	0.84	0.57	0.00
AFz	4	10.78	<0.001	0.24	0.72	0.57	0.00	1.30	0.25	0.01
Fz	4	13.08	<0.001	0.30	1.17	0.33	0.01	1.58	0.13	0.02
FCz	4	13.71	<0.001	0.29	2.80	0.03	0.06	1.18	0.31	0.01
Cz	4	13.69	<0.001	0.29	3.37	0.01	0.07	0.71	0.68	0.00
CPz	4	6.29	<0.01	0.14	1.96	0.10	0.03	0.57	0.81	0.00
Pz	4	5.77	<0.01	0.13	0.88	0.47	0.00	0.83	0.57	0.00
POz	4	9.21	<0.001	0.21	0.32	0.86	0.00	1.21	0.29	0.01
Oz	4	15.67	<0.001	0.32	1.97	0.10	0.03	1.26	0.27	0.01

### Statistics

Statistical analysis was conducted using the software JASP (0.8.4, JASP Team (2018), Amsterdam, Netherlands). For analysis of the data acquired, the basic parameter for comparison is the mean. The mean was tested with a repeated analysis of variance (ANOVA). A *p*-value < 0.05 was deemed statistically significant. Thus, the five different tinted lenses were compared for statistical significance.

The mean values of the responses to each picture were calculated. These mean values of the LPP (500–1,500 ms) were extracted from the EEG data, these mean values of ISCR and tonic SCR were extracted from the GSR data and the mean LF/HF ratio was extracted from the HRV data. To determine the effect of factor tint and factor valence, the data was entered in a two-way ANOVA. *Post hoc* analysis was performed pairwise with Bonferroni correction for multiple comparisons.

## Results

### Preference

The liking for the green-tinted lens revealed with 74.2% the highest, followed by blue- with 51.6%, then red- with 48.4% and finally yellow-tinted lenses with 29.0%. Dislikes were most for red- with 35.5%, then yellow- with 25.8%, blue- with 3.2% and green-tinted lenses with zero percentage. Blue- and yellow-tinted lenses were equally rated as neutral with 45.2%, green- with 25.8% and red-tinted lenses with 16.1%.

### Heart Rate

In the HRV, the LF/HF ratio showed no significant effect for factor tint (*p* = 0.63) or factor valence (*p* = 0.66). Interestingly, the interaction between tint and valence became significant (*p* < 0.05) in this repeated ANOVA.

### Skin Conductance

The ANOVA of the phasic activity revealed a significant main effect for valence (*p* < 0.05), but not for tint (*p* = 0.78) or interaction (*p* = 0.60). Pairwise *post hoc* testing with Bonferroni correction showed that ISCR was significantly larger for unpleasant pictures compared to neutral pictures (*p* < 0.05), but not between pleasant and neutral pictures (*p* = 0.17) and not between pleasant and unpleasant (*p* = 0.69).

The ANOVA of the tonic activity returned a main effect for tint (*p* < 0.01), but not for valence (*p* = 0.56) or interaction (*p* = 0.34). A pairwise Bonferroni-corrected *post hoc* comparison revealed a significant elevation of only red-tinted lenses compared to none (*p* < 0.01).

### Late Positive Potential

**Figure 3:**
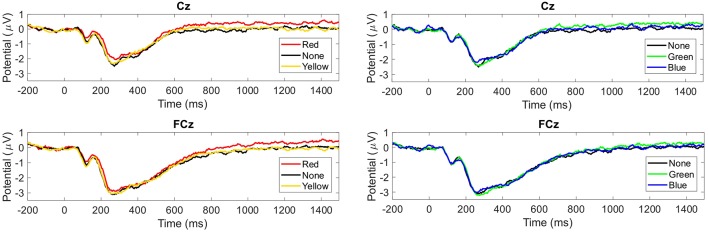
Group mean event-related potentials (ERPs) evoked by tint condition. Upper graphs from Cz and lower graphs from FCz. Left: red-, none- and yellow-tinted lens. Right: none-, green- and blue-tinted lens. Time in milliseconds from picture onset is plotted.

**Figure 4:**
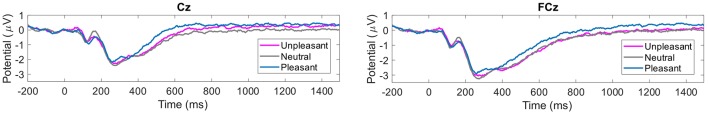
Grand average of the ERPs for each valence condition. Left: Cz. Right: FCz. Time in milliseconds from picture onset is plotted.

The grand average of the ERP revealed a component, which differentiated for the factors of valence and tint. For tint, the grand averaged ERP is illustrated in Figure 3 and for valence in Figure 4. This component was similar to the LPP. The LPP amplitude resembled a plateau in Figure 3 and was larger when participants wore red- or green- than none-tinted lenses. In contrast, a similar increase was not visible in blue- or yellow- tinted lens conditions, see Figure 3. Separately, there appeared to be a difference between pleasant and neutral valence pictures in the LPP component, see Figure 4. Interestingly, only in Cz the LPP was elevated for unpleasant conditions compared to neutral condition, see Figure 4 left, but not in FCz, see Figure 4 right.

An ANOVA of the mean voltage potential of Cz returned significant main effects for valence (*F*_(2,60)_ = 13.7, *p* < 0.001) and tint (*F*_(4,120)_ = 3.4, *p* < 0.05), but not their interaction (*F*_(8,240)_ = 0.71, *p* = 0.68) see Table 2. For FCz, which was calculated by the mean of FC1 and FC2, the ANOVA of the mean voltage potential again revealed a significant effect for valence (*p* < 0.001) and tint (*p* < 0.05), but not their interaction (*p* = 0.31) see Table 2.

**Table 2:** ANOVA of LPP (500–1,500 ms) in detail.

	Cz					FCz				
	*df*	Mean Square	*F*	*p*	*ω*^2^	*df*	Mean Square	*F*	*p*	*ω*^2^
Tint	4	2.98	3.37	0.01	0.07	4	1.64	2.80	0.03	0.06
Valence	2	6.15	13.69	<0.001	0.29	2	6.27	13.71	<0.001	0.29
Tint * Valence	8	0.29	0.71	0.68	0.00	8	0.37	1.18	0.31	0.01

For further central electrodes, the main effect for tint was significant in FCz and Cz. The electrodes Fpz, AFz (mean of AF3 and AF4), Fz, Pz and POz showed no significant effect for tint, whereas CPz and Oz showed a trend with *p* = 0.10, see Table 1. Furthermore, no significant interaction effect was found between tint and valence.

#### Valence

Pairwise Bonferroni-corrected *post hoc* testing for valence revealed that pleasant (*p* < 0.001) and unpleasant (*p* < 0.05) pictures elicited larger LPPs compared to neutral pictures in Cz. For FCz, the LPP difference between neutral and pleasant was significant (*p* < 0.001). For Cz and FCz, pleasant pictures elicited significant larger LPPs compared to unpleasant pictures (*p* < 0.05; *p* < 0.001). For Cz, LPP of unpleasant pictures were significantly larger than neutral (*p* < 0.05), but not in FCz (*p* = 1.00). This reduction of LPP to unpleasant relative to pleasant pictures appears in an overlapping ERP response to unpleasant and neutral pictures in FCz, see Figure 4 right.

#### Tinted Lens

Regarding single tinted lenses, the *post hoc* Bonferroni-corrected pairwise comparison revealed a significant difference between red-tinted lens and no filter condition (*p* < 0.001) as well as between red- and yellow-tinted lens (*p* < 0.05) in terms of LPP. Equivalent statistical results for FCz are listed in Table 3. In addition, in Cz the difference in LPP between green- and none-tinted lens was found to be significant (*p* < 0.05), see Table 3.

**Table 3:** Pairwise *post hoc* comparisons with Bonferroni-Correction for Tint.

		Cz	FCz
		*p*_bonf_	*p*_bonf_
None	Blue	1.000	1.000
	Red	<0.001	0.005
	Yellow	1.000	1.000
	Green	0.049	0.561
Red	Yellow	0.026	0.026
	Green	1.000	0.789
	Blue	0.166	0.197
Blue	Green	1.000	1.000
	Yellow	1.000	1.000
Green	Yellow	1.000	1.000

**Figure 5:**
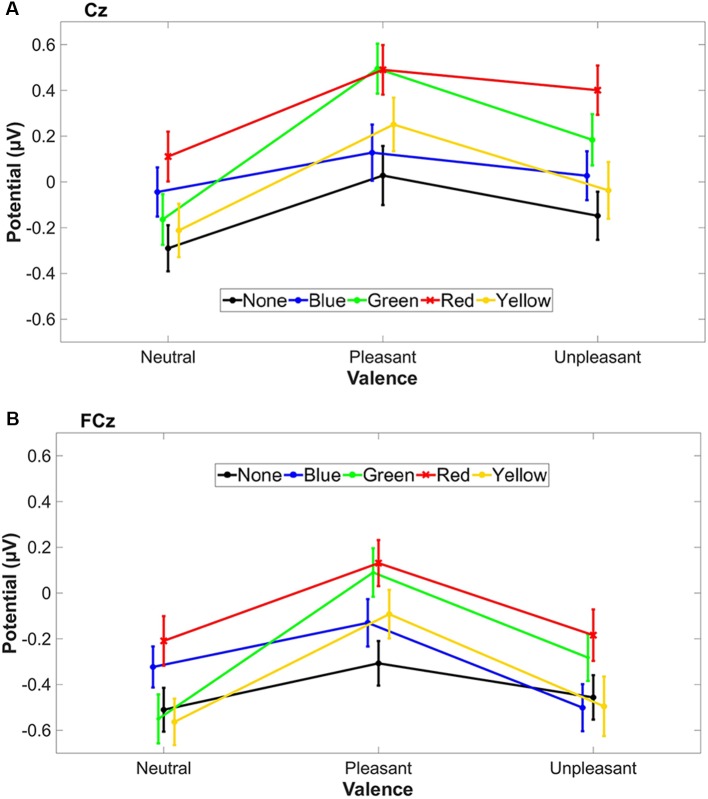
The standard error of the mean (SEM) and the mean LPP amplitude (500–1,500 ms) of tinted lens conditions are plotted for each stimulus valence condition for Cz **(A)** and FCz **(B)**. Valence conditions are connected with a line for better visualization.

All three valence conditions with red-tinted lenses showed the highest LPP mean values (Figures 5A,B), whereas green-tinted lens reached a similar level in the pleasant condition compared to red.

## Discussion

In this study, the effect of color-tinted lenses was investigated on a brain’s and autonomic response to emotional events. Increased autonomic arousal was found to unpleasant pictures and enhanced cortical reaction to pleasant pictures as well as to red-tinted lenses. This suggests that unintentional responses to unpleasant events are modulated at the cortical level; this regulation can be achieved by tinted lenses. Since LPP changed mainly to pleasant and surprisingly not to unpleasant events, we expected a positive influence of tinted lenses on affective processing. Nevertheless, we found no interaction between tint and valence. This means that the effect of color-tinted lenses on LPP can be independent of affective processing. However, several key questions remain:

(1) Which factors besides tinted lenses could also differentiate the reactions to pleasant and unpleasant events? How do GSR and HRV contribute under the influence of tinted lenses?

Participants reacted to emotional pictures with an increased LPP, especially to pleasant pictures in EEG and an elevated ISCR to unpleasant pictures. This agrees only partly with previous findings that emotionality is encoded in higher LPP and skin conductance (e.g., Lang et al., 1993; Cuthbert et al., 2000). We found that this autonomic connection to affective processing occurs also under the influence of tinted lenses in the presence of unpleasant pictures. However, pleasant pictures shifted the LPP even higher than unpleasant images with all tinted lens conditions, which was not reflected in the ISCR. We assume that tinted lenses in general lead to a more positive level by enhancing pleasant and reducing unpleasant impressions on the cortical level. An alternative explanation might be that this enhancement of pleasant pictures is an effect of the differentiation that pleasant pictures involve other brain areas than unpleasant pictures (Liu et al., 2012), leading to an earlier positive shift of pleasant compared to unpleasant pictures (Cuthbert et al., 2000). This earlier positive shift of pleasant pictures is also observed, see Figure 4. Nevertheless, this earlier onset of pleasant pictures does not necessarily lead to an increased LPP amplitude. In the literature, contradictory results are reported. Most previous studies did not find an amplitude difference in LPP between pleasant and unpleasant pictures (see review Schupp et al., 2000; Olofsson et al., 2008), however some did (e.g., Cuthbert et al., 2000; Carretié et al., 2006). Additional physiological measurements from heart rate revealed no further insights. Although others found that HRV can reflect human emotion (Kop et al., 2011), but with IAPS pictures only with strong emotional stimuli (Choi et al., 2017), we found no effect of emotional pictures on HRV for tint and valence separately, but for their interaction.

(2) Can the effect of red tinted lenses be explained by the arousal theory or the color-in-context theory?

It could be shown for the first time that red-tinted lenses elevated cortical components such as LPP, specifically in central and fronto-central brain regions, and autonomic levels such as tonic SCR during emotional and neutral picture presentation. This enhancement by red-tinted lenses might come from visual attention, which is captured and sustained by emotionally arousing pictures (Hajcak and Olvet, 2008). Whereas, the P300 indicates a phasic increase in attention to task-relevant stimuli, the LPP seems to follow the sustained increase in attention toward intrinsically motivating events (Hajcak et al., 2010). The red tint condition showed a higher LPP, even for neutral stimuli. This general increased LPP for red might, therefore, indicate a stronger emotional response of the perceived stimuli which is independent of the valence because we found no interaction between valence and tint. The findings from color psychology studies support a red enhancing effect (Goldstein, 1942; Wexner, 1954). Two theories try to explain the red enhancing effect: first the arousal theory of color (Wilson, 1966) and the color-in-context theory (Buechner and Maier, 2016). The arousal theory is based on Wilson’s hypothesis that wavelength vs. arousal shows a U-shaped behavior, that is, colors composed of extremer wavelengths are more arousing (Wilson, 1966). The color-in-context theory claims that the context, in which the color is perceived, affects the emotional response (Elliot and Maier, 2012). Regarding the color-in-context theory, it could be expected that colored lenses should influence only emotional picture presentation and not neutral pictures. For the red-tinted lens condition, the context of valence does not seem to matter, because the LPP was elevated in all three valence conditions. This effect contradicts the color-in-context theory. The color red gets an attentional function, when embedded in non-red context, highlighting the relevance and importance (Buechner et al., 2014) e.g., in red primed angry and happy human facial expressions. This coloration effect of red is not applicable when tinted lenses are worn because a tinted lens colors the entire scenery and not only a single object. This difference between isolated and global coloration might explain the discrepancy of our results to the color-in-context theory. Our findings are in line with the theory that red by itself is arousing. On cortical and autonomic level, the effect of red-tinted lenses can be independent of the three different valence conditions, because we found no interaction on both levels between tint and valence. Furthermore, the agreement to the arousal theory is supported by the evaluated preferences: only 16.1% of the participants rated red as neutral, but the majority of 83.9% assessed red as “not neutral” (48.4% likes + 35.5% dislikes). Participants may have already recognized that their arousal changed with red-tinted lenses immediately and they rated this change as “not neutral”—already prior to the main experiment and before the perception of emotional events. The high color preferences for green, blue and red are in line with the Ecological Valence Theory (Palmer and Schloss, 2010).

(3) What explains the LPP increase with red-tinted lenses besides psychological explanations?

There might be also an evolutionary explanation. Many animals such as birds show an increased activity during twilight i.e., during dawn and dusk (Palmgren, 1949). During this transitional phase of twilight, environmental stimuli change drastically and quickly i.e., the intensity of illuminance and its associated spectral colors (Stahlbaum et al., 1986). Usually during sunset or sunrise, longer reddish wavelengths are more present than during the daylight, mainly due to amplified Rayleigh scattering because light has to travel much farther through the atmosphere. At times of twilight, sensory areas of the brain are reduced, which facilitates visual detection performance (Cordani et al., 2018). It was shown that the LPP reflects a global inhibition in the visual cortex when emotional stimuli are processed (Brown et al., 2012). Taking these two findings hypothetically together and projecting to our findings, the red-tinted lens may trigger a system activating arousal, which could be involved in the regulation of the twilight activity. To prove this speculative idea and to support more insights, studies with tinted lenses and circadian activity are necessary in the future.

(4) Is there a chance that cortical effects of tinted lenses have nothing to do with hue?

It was shown before that red colored stimuli amplified the skin conductance responses compared to blue stimuli, as well as saturated compared to desaturated stimuli (Rajae-Joordens and Hanique, 2012). The authors suggest that the effect of red light derives from the perceived saturation difference among different colored light. While the luminance was controlled in our experiment, the saturation was not. It might be that the saturation of red-tinted lenses was elevated. Future experiments can address the saturation in tinted lenses. With colored light, it was shown that saturation and brightness of colors have an increasing effect on SCR, also the hue has affected the arousal, and most of all with red (Wilms and Oberfeld, 2018).

Furthermore, the results cannot be generalized to all age groups because we investigated a relatively narrow age group around ca. 26 years. A problem with older participants is that their lens gets yellow in parallel with cataract, which would add an additional color on top to the investigation. This means that transmittance of the crystalline lens decreases with age (Weale, 1988). Therefore, only a young population was included in this investigation. Besides tinted lens, a potential effect of red on arousal and cortical response must also be considered when visible light is filtered software-wise, specifically when the light source changes its spectrum. This happens in the so called “night shift” applications of modern devices like smartphones, tablets or monitors. Such applications reduce the short wavelength components to avoid circadian rhythm shifts. However, it could not be proven that changing color spectrum alone is changing the melanopsin suppression using the “night shift” function (Nagare et al., 2019). In addition to circadian rhythm, the implication of such activated “night shift” filters for emotional arousal and cortical enhancement may be important when watching photos and movies at night or studying at night. Our investigated LPP component, which is starting from 500 ms, belongs to later components (>300 ms) associated with memory encoding (Olofsson et al., 2008). A tinted lens that changes the LPP may, therefore, aid memory encoding. Therefore, further application of red-tinted lenses could be enhancing effects to memory as it has been shown that arousal is able to enhance long-term memory (Singh and Churchill, 1987). Green-tinted lenses increased LPP as well, but mainly in pleasant picture condition, which would strengthen the previously reported idea that green is associated with positive mood (Akers et al., 2012). Additionally, participants evaluated the green-tinted lens with the most likes (74.2%). However, the effect of green-tinted lenses can be also interpreted as independent since there was no significant interaction between tint and valence. If not independent, such a positive implication of green-tinted lens would be aligned with the headache reducing the effect of green light (Noseda et al., 2016). In future studies, it might be worth to investigate if green-tinted lenses have a similar headache reduction like green light. Interestingly, in all tinted lens conditions, the autonomic level changed to unpleasant pictures, whereas the cortical level mainly changed to pleasant pictures. A similar trend occurred for the red-tinted lens independent of the valence. Red-tinted lenses increased physiological basic arousal as well as cortical level. On a physiological level, red-tinted lenses are rather arousing independent of the emotional condition. Possible applications of a red-tinted lens could be to keep arousal level high e.g., during tiring or demanding work or watching pictures.

In summary, the findings indicate that the autonomic response to affective images is modulated at the cortical level of processing. Interesting, the use of red-tinted lenses has a similar influence, in that it also elevates LPP potentials. Thus, it is possible that the presence of red tints could increase the perceived valence of images. It should be noted that the current study does not reveal a significant interaction between the two factors in the LPP and ISCR. Hence, it is premature to believe that red tints moderate valence differentially. Nonetheless, the current results converge with known behavioral findings and subjective reports. Furthermore, we are confident that the results did not arise from low-level differences across color tints, such as contrast and luminance, which could have affected the visibility or spatial content of the images. Having isolated the influence of color tints to red, subsequent work could further investigate if these two factors might interact if the physical properties of red tints and subjective norms of valence intensity are parametrically manipulated. This would lend more statistical power to determine if the color red can directly moderate affective processing or if it introduces an independent influence on affective processing.

## Ethics Statement

This study was carried out in accordance with the recommendations of Institutional Review Board of the Medical Faculty of the University of Tuebingen with written informed consent from all subjects. All subjects gave written informed consent in accordance with the Declaration of Helsinki. The protocol was approved by the Institutional Review Board of the Medical Faculty of the University of Tuebingen.

## Author Contributions

All authors were involved in the interpretation and summarizing of the study, and their special contributions were the following: TS, LC and SW designed the experiment; TS and AS conducted the experiment; TS, LC and AS analyzed the data; SW was the principal investigator. All authors reviewed the manuscript.

## Conflict of Interest Statement

TS and AS are scientists at the University Tuebingen. LC is a scientist at the Max Planck Institute for Biological Cybernetics Tuebingen and at the Institute for Informatics Ludwig-Maximilians-University Munich. SW is employed by Carl Zeiss Vision International GmbH and is a scientist at the University Tuebingen. TS is employed by Dopavision GmbH.

## Footnotes

^1^Pleasant IAPS picture No.: 2208, 2250, 2260, 2501, 2560, 2650, 4611, 4617, 4640, 4653, 4659, 4666, 4687, 4694, 5621, 8041, 8080, 8090, 8116, 8120, 8161, 8180, 8200, 8280, 8300, 8320, 8330, 8370, 8380, 8400, 8420 and 8465

^2^Neutral IAPS picture No.: 2020, 2190, 2200, 2210, 2214, 2215, 2220, 2221, 2235, 2240, 2270, 2272, 2278, 2383, 2393, 2410, 2441, 2491, 2493, 2514, 2579, 2745.1, 2749, 2752, 2810, 2850, 2870, 2890, 3210, 5455, 7550 and 9210

^3^Unpleasant IAPS picture No.: 2120, 2205, 2520, 2590, 2691, 2730, 2750, 2800, 3015, 3030, 3053, 3100, 3170, 3180, 3181, 3400, 3500, 3530, 3550, 6210, 6211, 6212, 6821, 6834, 6838, 9041, 9250, 9254, 9341, 9405, 9800 and 9921
